# Bacterial Meningitis in Children With Sickle Cell Disease in Angola

**DOI:** 10.1097/INF.0000000000003581

**Published:** 2022-07-13

**Authors:** Tuula Pelkonen, Irmeli Roine, Luis Bernardino, Kirsi Jahnukainen, Heikki Peltola

**Affiliations:** From the * Pediatrics, University of Helsinki and Helsinki University Hospital; † New Children’s Hospital, Pediatric Research Center, Helsinki, Finland; ‡ Hospital Pediátrico David Bernardino, Luanda, Angola; § Faculty of Medicine, University Diego Portales, Santiago, Chile.

**Keywords:** meningitis, sickle cell disease, Africa, child, outcome

## Abstract

Sickle cell disease (SCD) was found in 10% of children with bacterial meningitis (BM) in Luanda, 5-fold more than in the general population. BM children with SCD versus BM children without SCD had higher inflammatory markers, more often had pneumococcal meningitis (71% vs. 39%), and either died (39% vs. 22%) or had a longer hospital stay (15 vs. 11 days).

Acute bacterial meningitis (BM) continues to be an important cause of child mortality and morbidity. With the introduction of protein conjugate vaccines, the incidence of BM has declined particularly in high-income countries. Globally, however, the number of reported BM cases has risen during the past decade.^1^

Sickle cell disease (SCD) is a collective term for genetic blood disorders caused by sickle hemoglobin (Hb). The most common form of SCD is sickle cell anemia (SCA), the homozygous state. The heterozygous carrier state or sickle cell trait (SCT) is almost always asymptomatic.^2,3^

The burden of SCA is greatest in sub-Saharan Africa and the highest frequency of the sickle cell allele, approximately 18%, is found in northern Angola.^3^ However, with increasing migration, SCA can be encountered everywhere in the world.^3^ In Luanda, the capital of Angola, 36,453 newborns were screened for SCA 2011 to 2013.^4^ Of these, 77.3% had normal Hb, 21.0% SCT and 1.5% SCA. In another study performed in northern Angola, of 848,848 children 6–36 months old, 23.9% had SCT and 1.9% SCA.^5^

It is estimated that mortality among children <5 years with SCD in Africa can be up to 90%.^2^ Infection is probably the most important cause of premature deaths in SCD.^2,6^ Children with SCD are at high risk of invasive bacterial infections and BM, especially infections caused by *Streptococcus pneumoniae* or *Haemophilus influenzae*.^2,6^

In the present study, our objectives were (1) to compare the prevalence of SCD in children with BM with that in general population^4,5^ and (2) to compare the etiology, clinical course and outcome of BM in children with and without SCD. Our hypothesis was (1) that SCD would be more common in children with BM than in the general population and (2) that BM would be more severe in children with SCD than in children without SCD.

## METHODS

Hospital Pediátrico David Bernardino is a tertiary hospital in Luanda. An ambulatory sickle cell clinic operates at the hospital and provides the children with SCD a 13-valent pneumococcal conjugate vaccine (PCV13) and penicillin prophylaxis. In Angola, “Penta” vaccination (including *H. influenzae* type b) was launched in 2006 and PCV13 in 2013. Estimates for 3 doses of diphtheria-tetanus toxoids-pertussis vaccine coverage in Angola range from 57% to 84%, while estimates for PCV13 vaccination rates range from 59% to 82%.^7^

This was a secondary analysis of 2 prospective treatment trials in Hospital Pediátrico David Bernardino. The first trial recruited patients 2005 to 2008 and the second trial 2012 to 2017.^8,9^ The studies were approved by the local Ethics Committee. The children were enrolled after their guardian´s informed consent was obtained. The children were between 2 months and 15 years old and presented with signs suggestive of BM. BM was confirmed if a child with symptoms and signs of BM had (1) bacteria detected in cerebrospinal fluid (CSF), (2) positive blood culture or (3) 2 supporting laboratory criteria. All patients received cefotaxime for 7 days. Secondary antibiotic treatment was given, if considered clinically relevant and included some other antibiotic given beside cefotaxime and antibiotics given after 7 days (mostly ceftriaxone). Focal neurologic signs included ptosis, strabismus, facial paralysis, monoparesis and hemiparesis. Hearing was tested with brain evoked response audiometry. Details of the patients have been described previously.^8,9^

Newborn screening for SCD in Luanda started in 2011 and initially was not comprehensive.^4^ Our aim was to screen all children with BM for SCD with sickle Hb solubility testing and, if the test was positive, to perform Hb electrophoresis to identify cases of SCD. The sickle Hb solubility test is positive both in persons with SCT and SCD.

In the present analysis, we included children with BM and with known SCD or screening test result (see Figure, Supplemental Digital Content 1, http://links.lww.com/INF/E737). We excluded children who did not have a SCD screening. SCD was diagnosed in children with positive family history and/or characteristic symptoms with Hb electrophoresis, before or during admission. In the group of sickle cell screening-positive children, we included children who had positive screening and negative or unknown Hb electrophoresis and who did not have characteristic symptoms of SCD. Children with negative screening formed the group of children without SCD.

All data were computed and analyzed using JMP Pro 14.1.0 (SAS Institute, Inc, Cary, NC) for Windows. Contingency analysis, Pearson χ^2^ test and 1-way analysis of variance were used, as appropriate. To examine if SCD was an independent predictor of death, multivariate analysis was performed with known predictors of death, namely Glasgow Coma Score <13, ill before arrival > 5 days, weight-for-age Z score < –3 and seizures before or at arrival.

## RESULTS

Of the 1098 children included in the original BM studies, 595 (54%) had known SCD or screening test result and were included in the present analysis (see Figure, Supplemental Digital Content 1, http://links.lww.com/INF/E737). Of the 595 children, 57 (10%) had SCD, 143 (24%) had positive sickle cell screening and 395 (66%) had negative screening. If we consider the results of neonatal screening in Luanda,^4^ we estimate that 4 screening-positive children had SCD and other 139 had SCT. Of 174 children with pneumococcal meningitis, 34 (20%) had SCD, 40 (30%) had positive screening and 94 (54%) had negative screening. SCD diagnosis was known before admission in 51 of 57 (89%) children and was made at admission in 6 children (11%).

Table 1 shows characteristics of children with SCD or negative screening test (without SCD). The etiology of BM in SCD patients was *S. pneumoniae* in 71%, *H. influenzae* in 25%, *Staphylococcus aureus* in 2% and unidentified Gram-negative bacterium in 2%. In comparison, in children without SCD, the causative bacteria were *S. pneumoniae* in 39%, *H. influenzae* in 33%, *Neisseria meningitidis* in 18% and other bacteria in 11%.

**TABLE 1.** Characteristics of Angolan Children With Bacterial Meningitis and Sickle Cell Disease or Negative Sickle Cell Screening

Variable	All*	Sickle Cell Screening Negative	Sickle Cell Disease	*P*
No. of patients	452	395	57	
Demographics and history of illness				
Female sex	209/452 (46)	184/395 (46)	25/57 (44)	0.70
Age, yr	1.6 (0.6–4.3)	1.5 (0.6–4.5)	1.6 (1.1–2.8)	0.20
Number of children in the household	3 (2–4)	3 (2–4)	2 (2–4)	0.059
Running water at home	56/468 (12)	46/411 (11)	10/57 (18)	0.17
Electricity at home	259/468 (55)	217/411 (53)	42/57 (74)	0.003
Weight-for-age Z score	–1.37(–2.3 to –0.51)	–1.40 (–2.36 to –0.52)	–1.28 (–2.25 to –0.47)	0.75
Ill before admission, d	5 (3–7)	5 (3–7)	5 (3–8)	0.33
Previous antibiotics	180/428 (42)	152/374 (41)	28/54 (52)	0.12
Cerebrospinal fluid analysis				
CSF-leukocytes, /mm^3^	720 (143–2275)	573 (132–2031)	1200 (461–3730)	0.008
CSF-glucose, mg/dL	18.2 (8.3–35.0)	19.6 (9.1–36.3)	9.7 (5.3–21.4)	0.0008
CSF-protein, mg/dL	177 (102–258)	169 (101–265)	184 (121–238)	0.89
Causative bacteria	291	243	48	0.0001
*Haemophilus influenzae*	92/291 (31.6)	80/243 (33)	12/48 (25)	
*Streptococcus pneumoniae*	128/291 (44.0)	94/243 (39)	34/48 (71)	
*Neisseria meningitidis*	43/291 (14.8)	43/243 (18)	0/48 (0)	
Other bacteria	28/291 (9.6)	26/243 (11)	2/48 (4)	
Blood tests				
Hemoglobin day 1 (–2), g/dL	7.5 (6.3–9.0)	7.9 (6.6–10.3)	5.7 (4.6–6.6)	<0.0001
Hemoglobin day 4 (± 1), g/dL	8.0 (7.1–9.0)	8.1 (7.1–9.2)	6.5 (5.5–8.2)	0.002
C-reactive protein, day 1 (–2), mg/L	147 (69–161)	136 (61–161)	161 (103–161)	0.018
C-reactive protein, day 4 (± 1), mg/L	87 (47–155)	86 (36–133)	120 (75–161)	0.057
Erythrocyte sedimentation rate, mm/h	80 (49–109)	74 (46–105)	100 (78–119)	0.012
B-leukocytes × 10^9^/L	13.9 (8.6–19.6)	13.0 (8.5–18.7)	20.5 (14.1–34.3)	0.0006
B-thrombocytes × 10^9^/L	292 (176–449)	301 (177–459)	229 (163–356)	0.12
Malaria thick film positive	132/431 (31)	120/377 (32)	12/54 (22)	0.15
HIV positive	28/387 (7)	26/351 (7)	2/36 (6)	0.68
Symptoms and signs				
Glasgow Coma Score	12 (8–15)	12 (8–15)	12 (8–15)	0.86
Seizures before or at arrival	204/450 (45)	169/391 (43)	32/56 (57)	0.080
Seizures at ward	257/452 (57)	220/395 (56)	37/57 (65)	0.19
Focal neurologic signs	104/446 (23)	83/390 (21)	21/56 (38)	0.007
Other focus of infection	257/450 (57)	220/393 (56)	37/57 (64)	0.21
Dyspnea	195/451 (43)	163/394 (41)	32/57 (56)	0.035
Treatment in hospital				
Secondary antibiotic treatment	157/451 (34)	131/395 (33)	26/56 (46)	0.051
Anti-convulsion medication	265/452 (59)	226/395 (57)	39/57 (68)	0.11
Blood transfusion during hospital stay	150/452 (33)	110/395 (28)	40/57 (70)	<0.0001
Supplementary oxygen	214/452 (47)	180/395 (46)	34/57 (60)	0.047
Malaria treatment	263/452 (58)	235/395 (59)	28/57 (49)	0.14
Outcome				
Length of hospital stay, d	11 (9–17)	11 (9–16)	15 (11–22)	0.0003
Death	107/452 (24)	85/395 (22)	22/57 (39)	0.005
Severe neurologic sequelae†	51/343 (15)	47/308 (15)	4/35 (11)	0.55
Deafness‡	34/323 (11)	30/292 (10)	4/31 (13)	0.65

* Data presented as median (interquartile range) or n/N (%).

† Blindness, quadriplegia/paresis, hydrocephalus requiring a shunt or severe psychomotor retardation.

‡ Hearing threshold ≥80 dB.

On admission, children with SCD had significantly higher CSF-leukocyte count and lower CSF-glucose than children without SCD. The inflammatory markers C-reactive protein, erythrocyte sedimentation rate and blood leukocyte count were also significantly higher in children with SCD compared with children without SCD.

Median Hb on day 1 (–2) was 5.7 g/dL in children with SCD and 7.9 g/dL in children without SCD. On day 4 ± 1, the respective Hb values were 6.5 g/dL and 8.1 g/dL. Blood transfusion, mostly performed on the day of admission, was given to 70% of children with SCD and to 28% of children without SCD. Children with SCD had dyspnea and received supplementary oxygen significantly more often than children without SCD. Focal neurologic signs were more common in children with SCD than in children without SCD.

Deaths occurred in 39% of children with SCD and in 22% of children without SCD. Of children with pneumococcal meningitis, 47% children with SCD and 18% of children without SCD died (*P* = 0.0009). In multivariate analysis with known predictors of death (see Table, Supplemental Digital Content 2, http://links.lww.com/INF/E738), SCD increased the odds for death 2.51-fold (95% confidence intervals, 1.33–4.74; *P* = 0.005). Of the children who survived, children with SCD recovered slower and required a median of 15 days of hospital stay compared with 11 days for children without SCD. Sequelae registered among 35 survivors with SCD included severe psychomotor retardation (3 children), quadriplegia (1 child), moderate psychomotor retardation (4 children) and hemiparesis (3 children). There were no significant differences between the groups in sequelae.

## DISCUSSION

SCD was found in 10% of children with BM in Luanda, manyfold more than in the general population. Comparison of the prevalence of SCD in the study children and in general population revealed that the estimated risk of a child with SCD acquiring BM was ≥5 times higher than that in other children and the risk for pneumococcal meningitis was ≥10 times higher. In the present study, malnutrition, and HIV-positivity, known factors to increase the risk of acquiring BM, were found with equal frequency in children with or without SCD. Children with SCD and BM had higher inflammatory markers, more often had pneumococcal meningitis, and either died or had a longer hospital stay than other children.

The proportion of children with SCD in this study is comparable with a study in Kenya, where 9% of children with bacteremia and BM had SCD.^10^ In a review including 3 BM studies from Africa before introduction of conjugate vaccines, the pooled odds of SCD was 20 for all-cause BM, 25 for pneumococcal meningitis and 9 for *H. influenzae* meningitis.^11^

In this study, the risk of death of children with SCD in BM was higher than that of children without SCD and the difference was even clearer in pneumococcal meningitis. This contrasts with the traditional belief that the risk of death in patients with BM and SCD does not exceed that of other patients.^6^ In the United States, children with SCD and invasive pneumococcal disease were more likely to be hospitalized and more likely to die than children without risk factors.^12^

A study in Luanda revealed that newborn screening is also feasible in sub-Saharan Africa.^4^ When the condition is diagnosed, interventions such as vaccinations and penicillin prophylaxis can be introduced to decrease the risk of invasive infections and BM.^2,6^ Hydroxyurea therapy reduces mortality and infections in children with SCD.^13^

There are some limitations to this study. Sickle cell status was known in only 54% of the children. Limited resources prevented doing screening tests immediately at admission and Hb electrophoresis for all children. Characteristics of BM among children with SCT could not revealed. The strengths of our study, in addition to the large number of patients, were standardized diagnosis, treatment and monitoring of meningitis and meticulous collection of clinical data.

To conclude, the risk of BM and specifically of pneumococcal meningitis was higher in children with SCD than in the general population. BM in children with SCD is a devastating disease with high mortality. Efforts should be made for early diagnosis of SCD and for prevention of BM.

## ACKNOWLEDGMENTS

The authors thank all our colleagues, study nurses and laboratory technicians in Luanda and Finland.

## Supplementary Material


