# Supplementary material for: Bacterial Meningitis in Children With Sickle Cell Disease in Angola
Source: Pediatr Infect Dis J. 2022 Jul 13;41(8):e335–8. doi: 10.1097/INF.0000000000003581 (PMC9281509; doi:10.1097/INF.0000000000003581)
Supplement: Supplementary file 1 [file inf-41-e335-s001.pdf]

SUPPLEMENTARY FIGURE 1.

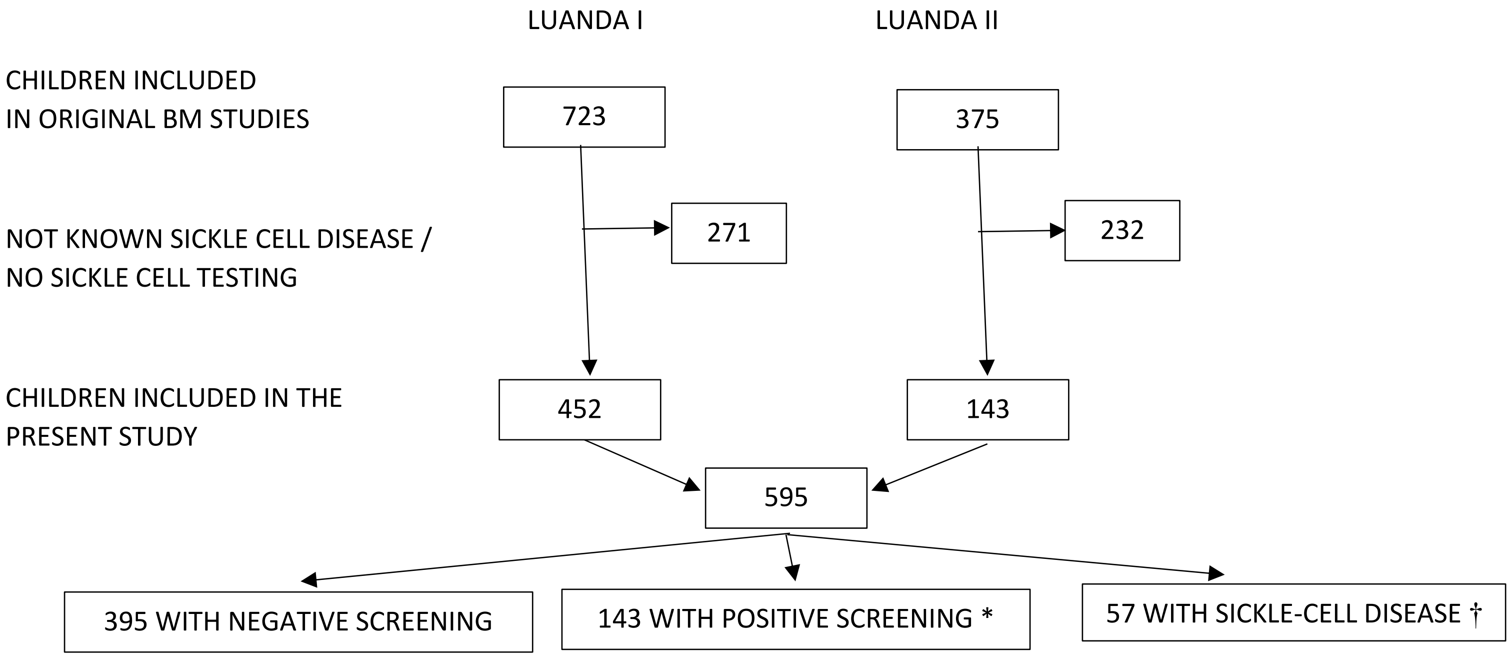

*Children with positive screening test and negative or unknown Hb electrophoresis result and without characteristic symptoms of sickle-cell disease.

† Children with family history and/or characteristic symptoms and Hb electrophoresis confirming the diagnosis of sickle-cell disease.
