# Supplementary material for: Bacterial Meningitis in Children With Sickle Cell Disease in Angola
Source: Pediatr Infect Dis J. 2022 Jul 13;41(8):e335–8. doi: 10.1097/INF.0000000000003581 (PMC9281509; doi:10.1097/INF.0000000000003581)
Supplement: Supplementary file 2 [file inf-41-e335-s002.docx]

| **Supplemental Digital Content 2.** Multivariate analysis of factors associated with death in children with bacterial meningitis in Angola. | | |
| --- | --- | --- |
| Variable | OR (95% CI) * | *P* |
| Glasgow Coma Score <13 | 3.18 (1.84 – 5.50) | <0.0001 |
| Ill >5 days before arrival | 2.41 (1.50 – 3.88) | 0.0003 |
| Sickle-cell disease | 2.51 (1.33 – 4.74) | 0.005 |
| Weight for age Z-score < - 3 | 2.41 (1.30 – 4.48) | 0.005 |
| Seizures before or at arrival | 0.99 (0.59 – 1.66) | 0.97 |
| * Odds ratios and 95% confidence intervals. | | |
